# A Mobile App to Support Self-management of Chronic Kidney Disease: Development Study

**DOI:** 10.2196/29197

**Published:** 2021-12-15

**Authors:** Talar W Markossian, Jason Boyda, Jennifer Taylor, Bella Etingen, François Modave, Ron Price, Holly J Kramer

**Affiliations:** 1 Department of Public Health Sciences Parkinson School of Health Sciences and Public Health Loyola University Chicago Maywood, IL United States; 2 Department of Informatics and System Development Loyola University Chicago Chicago, IL United States; 3 Center for Innovation for Complex Chronic Healthcare Hines Veterans Administration Hospital Hines, IL United States; 4 Health Outcomes & Biomedical Informatics College of Medicine University of Florida Gainesville, FL United States; 5 Department of Medicine Loyola University Chicago Maywood, IL United States

**Keywords:** chronic kidney disease, mobile app, self-management, mHealth, mobile apps, digital health, kidney disease, smartphone

## Abstract

**Background:**

Chronic kidney disease (CKD) is a common and costly condition that is usually accompanied by multiple comorbidities including type 2 diabetes, hypertension, and obesity. Proper management of CKD can delay or prevent kidney failure and help mitigate cardiovascular disease risk, which increases as kidney function declines. Smart device apps hold potential to enhance patient self-management of chronic conditions including CKD.

**Objective:**

The objective of this study was to develop a mobile app to facilitate self-management of nondialysis-dependent CKD.

**Methods:**

Our stakeholder team included 4 patients with stage 3-4 nondialysis-dependent CKD; a kidney transplant recipient; a caretaker; CKD care providers (pharmacists, a nurse, primary care physicians, a nephrologist, and a cardiologist); 2 health services and CKD researchers; a researcher in biomedical informatics, nutrition, and obesity; a system developer; and 2 programmers. Focus groups and in-person interviews with the patients and providers were conducted using a focus group and interview guide based on existing literature on CKD self-management and the mobile app quality criteria from the Mobile App Rating Scale. Qualitative analytic methods including the constant comparative method were used to analyze the focus group and interview data.

**Results:**

Patients and providers identified and discussed a list of requirements and preferences regarding the content, features, and technical aspects of the mobile app, which are unique for CKD self-management. Requirements and preferences centered along themes of communication between patients and caregivers, partnership in care, self-care activities, adherence to treatment regimens, and self-care self-efficacy. These identified themes informed the features and content of our mobile app. The mobile app user can enter health data including blood pressure, weight, and blood glucose levels. Symptoms and their severity can also be entered, and users are prompted to contact a physician as indicated by the symptom and its severity. Next, mobile app users can select biweekly goals from a set of predetermined goals with the option to enter customized goals. The user can also keep a list of medications and track medication use. Our app includes feedback mechanisms where in-range values for health data are depicted in green and out-of-range values are depicted in red. We ensured that data entered by patients could be downloaded into a user-friendly report, which could be emailed or uploaded to an electronic health record. The mobile app also includes a mechanism that allows either group or individualized video chat meetings with a provider to facilitate either group support, education, or even virtual clinic visits. The CKD app also includes educational material on CKD and its symptoms.

**Conclusions:**

Patients with CKD and CKD care providers believe that a mobile app can enhance CKD self-management by facilitating patient-provider communication and enabling self-care activities including treatment adherence.

## Introduction

Chronic kidney disease (CKD) affects 15% of the US population and one out of every three US adults is at risk for developing CKD during their lifetime [1]. Proper management of CKD can delay or prevent kidney failure and help mitigate cardiovascular disease risk, which increases as kidney function declines [2]. However, the complexity of CKD management can be overwhelming for patients owing to multiple dietary restrictions, high pill burden, and inadequate disease education. In addition, CKD is usually accompanied by multiple comorbidities including type 2 diabetes, hypertension, and obesity [1,3], and individualized therapies to manage each comorbidity may not be concordant, which may frustrate the patient. Moreover, patients with CKD who receive discordant treatment recommendations owing to conditions that potentially complicate CKD management—for example, health failure or cancer—or because of having multiple medication prescribers have higher risk of health care usage and mortality [4].

Disease self-management is a recognized intervention for improving health status for individuals with chronic conditions [5,6]. While definitions of self-management are heterogeneous [7], the intervention of self-management shifts responsibilities from providers to patients who guide their care in partnership with health care providers [8,9]. Self-management may consist of two overarching domains of care: health care and everyday life [10,11]. Self-management of health care includes the interdependent dimensions of (1) communication between patients and providers, (2) partnership in care, (3) adherence to treatment regimens, (4) self-care activities, and (5) self-care self-efficacy defined as an individual’s confidence in disease self-management [12]. Self-management of everyday life involves achieving and maintaining “normality” in usual roles and functioning within the constraints set by living with a chronic condition and its emotional ramifications [10].

Improving self-management of both earlier and late stages of CKD has been associated with better health outcomes [13-18]. In one study, participation in a self-management support program comprising patient education, telephone-based support, and peer support was associated with lower rates of CKD progression [16].

The current pandemic has shown that self-management support needs to be readily accessible, in the hands of patients, and should not require extensive time away from home; this support could potentially be facilitated with a smartphone. In 2021, up to 85% of Americans owned a smartphone, including 61% of adults aged 65 years and older and 76% of persons with household incomes below US $30,000 [19]. In 2017, the number of health-related mobile apps exceeded 318,000, and the number of consumer wearable devices exceeded 340 [20]. In a 2013 survey of US adults, 69% reported keeping track of at least one health indicator, such as weight, diet, exercise routine, or symptoms, through their smartphone [21]. Information technology tools including mobile apps, web-based portals, and web-based educational or coaching interventions are increasingly being adopted to support disease self-management, and growing evidence links their usage to improved clinical outcomes [22-30]. However, mobile apps for CKD self-management targeted either specific clinical or health promotion domains [30,31], did not include the voices of patients with CKD in the app development process, did not focus on earlier stages of CKD, did not receive high ratings for clinical utility or usability, did not address patient safety concerns, and were developed by non-CKD care providers [32].

The overarching objective of this study was to develop a mobile app to enhance kidney disease self-management for nondialysis-dependent CKD. The novelty of our work is in the process of developing a mobile app that supports a holistic care approach and addresses both clinical care and health behavior promotion. In addition, the app is designed by persons with CKD and their providers, including nurses and pharmacists. In subsequent studies, we aim to assess the usability of the mobile app among patients with CKD and to describe patient and provider experiences, and the impact of the mobile app on improving patient activation and cardiovascular health.

## Methods

### Development Design

The Agile software development methodology guided the overall mobile app design [33]. We emphasized a co-design approach with continuous engagement of stakeholders at every stage of the development process [34] to deliver a product that is as user-friendly as possible. Our stakeholder team included 4 patients with stage 3-4 nondialysis-dependent CKD and a kidney transplant recipient, a caretaker, 2 primary care physicians, 3 PharmDs, a nephrologist, a cardiologist, a registered nurse from the nephrology clinic, a researcher in biomedical informatics (FM), 2 health services and CKD researchers (HK and TM), a systems developer, and 2 programmers. The providers in our key stakeholders’ team were recruited from the Loyola University Medical Center (LUMC) and patients were recruited from the LUMC’s nephrology clinic. The study was approved by the Loyola University Chicago’s institutional research board and patients and providers provided verbal informed consent prior to participation in the focus groups and interviews. All members of the stakeholder team owned a smartphone device.

### Data Collection

Two separate focus groups were facilitated by 2 members of the research team (TM and HK) and a programmer (JB). The first focus group included patients and a caretaker, and the second included the registered nurse and the PharmDs. A member of the research team (TM) conducted semistructured interviews with the additional stakeholders (2 primary care physicians, a nephrologist, and a cardiologist). Our focus group and semistructured interviews elicited a list of requirements and preferences regarding the content, features, and technical aspects of the mobile app. Focus groups and interview conversations were semistructured and completed using a guide that was based on CKD knowledge and self-management literature, as well as the mobile app quality criteria from the Mobile App Rating Scale [35]. One of the programmers (JB) reviewed the interview recordings and worked with the other programmer to build the mobile app (based on partial requirements). The programmer (JB) met with the research team biweekly, and intermediate versions of the app were presented to the research team for evaluation and feedback; this early version of the mobile app was then released to our stakeholders.

After the stakeholders had tested the mobile app, we conducted a second round of focus groups and semistructured interviews with stakeholders to elicit feedback about the second mobile app iteration and to identify needed modifications to optimize its usability and usefulness. Stakeholder recommendations for modifications were incorporated into a subsequent version of the mobile app. Because the COVID-19 pandemic began during the study period, the mobile app was modified to include information about COVID-19 infection and prevention. Focus group meetings and interviews were audio-recorded and transcribed verbatim for analysis. Focus groups lasted approximately 120 minutes, and semistructured interviews lasted approximately 35 minutes. The patients were provided lunch and a US $30 gift card to participate in the focus groups. In Table 1, we present the characteristics of the focus group and in-person interview participants (N=13).

**Table 1:** Characteristics of the participants of focus groups and in-person interviews (N=13). Patients with chronic kidney disease were aged 55-76 years.

Characteristics				Individuals, n
**Patients with chronic kidney disease**				5
	**Gender**			
		Female	1	
		Male	4	
	**Race**			
		White	3	
		Black	2	
	Hispanic ethnicity		1	
	**Chronic kidney disease stage**			
		Stage 3	2	
		Stage 4	2	
		Kidney transplant recipient	1	
**Chronic kidney disease care providers**				8
	PharmDs from population health		3	
	Registered nurse from the nephrology clinic		1	
	**Physicians**			
		Primary care	2	
		Nephrology	1	
		Cardiology	1	

### Data Analysis

We used NVivo (version 12, QSR International) qualitative data analysis software to support the analyses of all the focus group and interview data combined. Established qualitative analytic techniques were used, including the constant comparative method [36]. We deductively developed an initial code list a priori, which reflected categories of interest on the basis of elements of our conceptual model, and domains identified by our research team. Within each category, we then inductively developed additional codes and analyzed the text for themes and patterns. Coding entailed an iterative process where our codebook was revised to account for novel instances in the data. We identified key themes and concepts emerging from the data to generate meaningful categorization of the barriers and facilitators of CKD self-management and CKD app content and characteristics [36].

## Results

### Results Overview

The following themes were identified from the patient and provider stakeholder focus groups and interviews.

#### Theme A: Need for a Self-management App Specifically Designed for Patients With CKD

Patients described that CKD self-management was multifaceted, and they spoke about the challenges of having multiple apps on their phone, which track the various aspects of CKD self-management (ie, tracking their weight, diet, heart condition, blood pressure, and exercise). As one participant noted, “And what you need to have, which I have never seen, is some form of program that addresses people like us. Who have more than one disease problem.” They also described that they have not found an app they like to use to track their blood pressure. The patients in our focus group unanimously expressed that they thought a CKD mobile app could be helpful for disease self-management.

#### Theme B: Barriers and Respective Facilitators, When Identified, of CKD Self-management

Example extracts from the transcripts about barriers and respective facilitators of CKD self-management are presented in Table 2.

##### Code B.1: Healthy Lifestyle Is Challenging

Patients and providers agreed that maintaining a healthy lifestyle is challenging, and challenges are amplified in the setting of diabetes. Most providers acknowledged that poor dietary management may exacerbate CKD progression. Meanwhile, patients described the challenges of maintaining a healthy diet in the context of diabetes.

##### Code B.2: Early-Stage CKD Is Asymptomatic and Early Education Is Key

Most providers described that a major barrier for CKD self-management is the asymptomatic nature of the disease at early stages. Patients became aware of the seriousness of the condition only when the disease progressed to later stages, when it was too late to undo the damage caused to the kidneys.

##### Code B.3: Social Determinants of Health and the Importance of Addressing Them

Low income, lack of transportation, language, and lack of health literacy are barriers for maintaining healthy behaviors, and availability of translators and social work referrals for outpatient services can help underresourced patients.

##### Code B.4: Inability to Retain Information Within the Context of a Complicated Condition and Limited Duration of Provider Encounter

Patients described being overwhelmed with the information that the providers share with them during the encounter. However, providers discussed limiting the topics that they address during an encounter because of time constraints and the complexity and urgency of the patients’ conditions.

##### Code B.5: Need for Easily Accessible Nutrition and CKD Education for Patients and Primary Care Providers

Patients described their lack of knowledge about which foods and medications to avoid and the need for simple, easily accessible information. Providers described that patients did not understand how their bodily functions were related, and simple education about the association between high blood pressure, diabetes, and CKD would be effective. Providers also indicated that patients did not understand the medical indications for taking certain medications. Patients also need education on when to contact providers with regard to health concerns. For example, patients should be taught what blood pressure measurement threshold warrants an urgent visit to the doctor’s office or the emergency department. The primary care providers described that easily accessible guidelines for early-stage CKD management would be useful for their practice.

##### Code B.6: The Sequalae of Care Fragmentation and the Importance of Self-advocacy

Patients expressed frustration that they were receiving conflicting health information from different providers and that their providers did not communicate. Providers described similar situations. Providers also expressed frustration about the lack of coverage for medical nutrition therapy (MNT) for patients with early-stage CKD who do not have diabetes or heart disease, who most benefit from MNT to decelerate disease progression. One provider also describes difficulties in finding dietitians with expertise in nondialysis-dependent CKD. Patients described poor communication between different providers and felt this reduced the quality of the care they received.

##### Code B.7: CKD Is Stigmatized

Two patients spoke about hiding the CKD condition from their spouse and family to not worry them. One patient discussed the stigma associated with CKD and compared it with the stigma associated with obesity.

##### Code B.8: Feeling Stressed and Staying Positive

Patients spoke about feeling stressed and low on some days, and they described that stress impacted their blood glucose levels and “everything” else. Providers also spoke about the stressful nature of receiving a CKD diagnosis and the importance of maintaining positivity as a motivation for living with a CKD diagnosis.

##### Code B.9: Patient and Provider Shared Decision-making

Providers described their preference for a shared decision-making process to decide on a clinical course of action and mutual goal-setting as more desirable alternatives than providing authoritative advice. They also described that patients’ goals would be most effective when they are mutually set by the patients and providers.

**Table 2:** Barriers and respective facilitators of chronic kidney disease self-management.

Participant type			Example quotes
**Code B.1: Healthy lifestyle is challenging and is amplified in the context of diabetes**			
	Provider	*Honestly, I think the number one problem is weight and diet. That’s the most difficult thing that we deal with in primary care.* * If I see an obese, diabetic, hypertensive patient…I urge the patient to lose weight, and I tell them usually THAT’s the central goal in order to improve high blood pressure, diabetes and in turn slow down progression of CKD. *	
	Patient	*I’m trying to lose weight and when I don’t eat, all of a sudden my sugar drops.*	
**Code B.2: Early-stage chronic kidney disease is asymptomatic and early education is key**			
	Provider	*Patients can keep track of protein intake and their sodium intake, but, the majority of my patients are not going to when it’s an early disease that have no symptoms and they are not facing any imminent kidney issues.* *Because they are not having symptom doesn’t mean they are not at risk; so I think that point is really important to educate the patients on.*	
	Patient	*I think what really opened my eyes is when I came to that point where my creatinine, my GFR, got to 15 and the doctor said you’re going to need to go on dialysis. *	
**Code B.3: Social determinants of health and the importance of addressing them**			
	Provider	*I point patients to website to find information. I find that the National Kidney Foundation website has the most patient friendly information. The problem with the CKD population here at…is that health literacy is very low. And getting patients access to information is not always helpful because they are not able to process the information or retain it.*	
**Code B.4: Inability to retain information within the context of a complicated condition and limited duration of provider encounter**			
	Provider	*They come in with chest pain, I can’t talk about their kidneys at the same time. At each visit, you have to focus on what is important and you know try to at least touch on many of the other chronic problems as you can.*	
	Patient	*You’re hearing from this doctor, you’re hearing from that doctor. At the end of the day, you don’t remember any of that stuff.*	
**Code B.5: Need for easily accessible nutrition and chronic kidney disease education for patients and primary care providers**			
	Provider	*Some patients are confused about the correlation between their blood pressure and kidney disease.* *Sometimes it hard to stay on top of every specialist’s recent guidelines.* *I deal with every organ system.*	
	Patient	*Probably nobody here knew you can’t take Ibuprofen. And we are all probably taking it. Like I’m sore from exercising, I’m just going to pop a couple of Ibuprofen.* *I was doing everything right and then, what I come to find out is that my phosphorus kept staying high. Because salad dressing has a lot of phosphorus in it.*	
**Code B.6: The sequalae of care fragmentation and the importance of self-advocacy**			
	Provider	*Orthopedic doctor may put them on something that they might not recognize as being a nonsteroidal. It happens all the time.* *People who are sick enough that they are seeing specialists for other diseases whether its heart failure, or diabetes or kidney disease, if they are that sick that they need a specialist, then they are hooked up with nutritionists.*	
	Patient	*One doctor was giving me one thing, while the other gave me a medication that hurt my kidneys more.* *It’s up to you, the individual, to have those doctors communicate with each other.* *I suggested to him that I want to see a kidney specialist.*	
**Code B.7: Chronic kidney disease is stigmatized**			
	Patient	*I don’t see any reason why I have to stand up and say ‘I am fat’.*	
**Code B.8: Feeling stressed and staying positive**			
	Patient	*And from a personal level stress very much affect my glucose level.* *It (stress) affects everything. *	
	Provider	*Now that you’ve been diagnosed with kidney disease, it’s important to maintain positivity…look at that something motivational.*	
**Code B.9: Patient and provider shared decision-making**			
	Provider	*Probably comes out more as an authoritarian: ‘You have to monitor these kinds of things.’ Rather than trying to work out a sort of agreement with patients.*	

#### Theme C: Visually Appealing App That Is Easy to Use

Patients and providers spoke about wanting an app that is user-friendly, easy to use, and not complicated. Patients also mentioned their preference to self-navigate the app without asking the help of their children. As described in one interview, “You want to go and be able to do this by yourself… I don’t want to load too much on there and it starts to get complicated.” One patient mentioned her preference for an app in a “language” she understands, “Get something that is in a language that people can understand, and they can see that it’s going to help them if they work with it.”

#### Theme D: Peer Support and Scheduled Educational Group Meetings With a Provider

Patients discussed the benefits of sharing strategies for CKD management, including diets and recipes, and recommendations for doctors with their neighbors, and other patients with CKD. One patient reported, “Just about every day we congregate at my house and we talk about these things. We give each other ideas, and doctors, blah blah, blah. We tell them to go on men’s MD, researching so we help each other verbally.” In contrast, there was mixed enthusiasm for a peer support chat room embedded in the mobile app. Some patients were in favor of chatting with a peer through the app, especially on days when they were “feeling down,” while others were opposed to the idea. Providers were generally in favor of the concept of peer support. Nevertheless, both patients and providers supported the concept of periodic support groups focused on CKD education delivered by a provider.

### Mobile App Development

**Figure 1:**
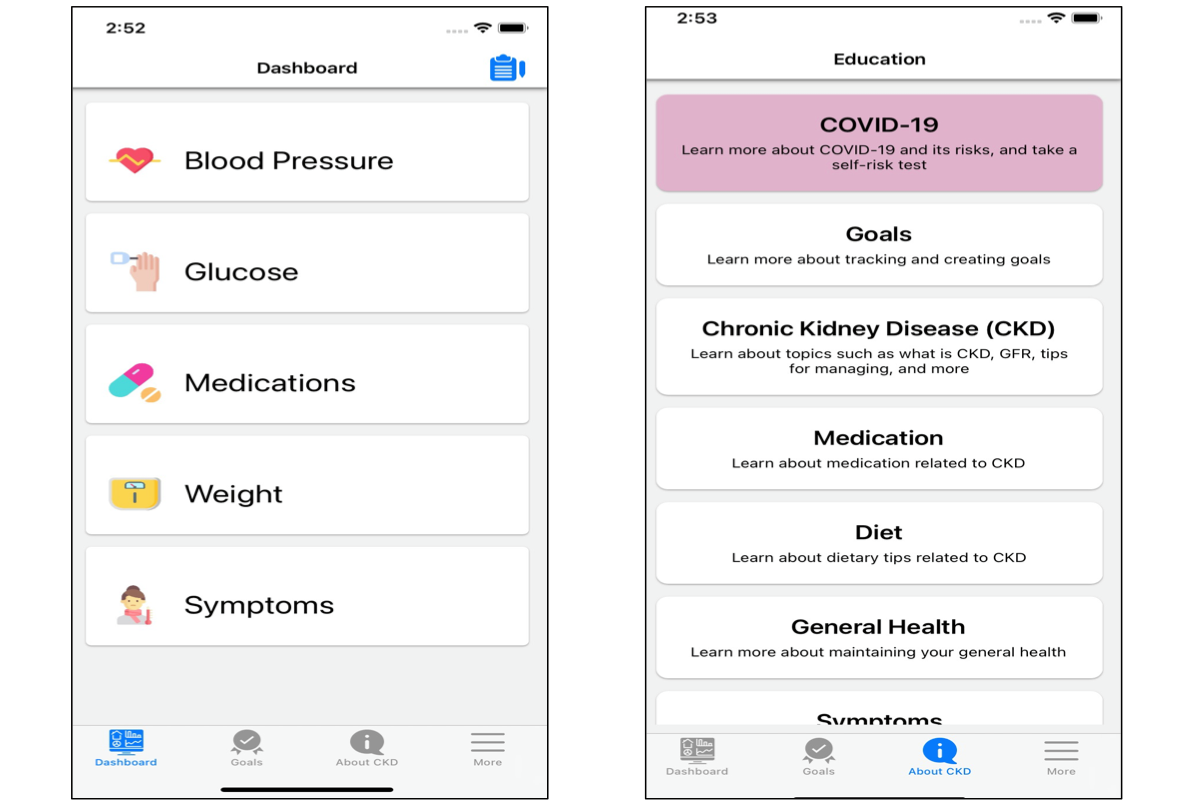
General and educational dashboards of the chronic kidney disease self-management mobile app.

Based on these discussions, we used an iterative process to develop a mobile app for CKD self-management. Textbox 1 shows the features included in the mobile app and the care management theme of the mobile app feature [10]. The developed mobile app has a simple user interface where the main functions are accessed through the app’s dashboard that provides access to measure-specific (eg, glucose and medications) panels (Figure 1). Each measure’s panel provides access to a series on controls that facilitate data collection on the targeted measure. The mobile app user can enter health data including blood pressure, weight, and blood glucose levels; symptoms and severity, where the user will receive a message indicating COVID-19 symptoms or symptoms requiring physician attention. Next, mobile app users can select biweekly goals from a set of predetermined goals with the option to enter customized goals. The user can also keep a list of medications.

Our app includes feedback mechanisms where in-range values for health data are depicted in green and out-of-range values are depicted in red. Certain predetermined goals are linked to biometric data entered by the user and would indicate green if the user met the goal. We ensured that data entered by patients could be downloaded into a user-friendly report that could be emailed or uploaded to an electronic health record, such as Epic, to facilitate communication with providers. The mobile app user can determine which data will be included in the report and dates included. The mobile app also includes a mechanism to facilitate either group or individualized video chat meetings with a provider to facilitate either group support or education or even virtual clinic visits. The CKD app also included educational material on CKD and its symptoms and educated patients on when to contact a provider. The app utilizes native mobile app controls and design principles. The app is implemented with simple language and a large font. The mobile app is available for iOS devices, and we are developing a version for Android devices. Additional details about the mobile app are presented in Textbox 1.

**App characteristics aligned with the self-management of health care dimensions. Since the dimensions of self-management of health care are interdependent, we represented the features of the app under the most relevant dimension.**

**Communication between patients and caregivers**

- Downloadable report of health indicators to share with providers (patients can customize which health indicators to include in the report, along with the monitoring time frame for each indicator) potentially include the following:
  Log of blood pressure with dates
  Log of weight with dates
  Log of symptoms with dates
  Log of biweekly goals with dates
  Log of medications currently taking
- Disclosure that the app is a tool to support self-management and will not substitute medical advice or replace physician consultation
- Prompts to contact their provider when entering symptoms which are considered emergent

**Partnership in care**

- Education content regarding the following:
  Chronic kidney disease: how kidneys function, recognizing symptoms associated with disease progression, managing blood pressure, medications and supplements to avoid (including herbals, vitamins, and minerals), blood glucose level and implications, chronic kidney disease–related blood analysis
  Nutrition and recommended diets for patients with chronic kidney disease
  The concept of self-management and creating SMART (Specific, Measurable, Achievable, Realistic, and anchored within a Time Frame) goals
  Anxiety and depression
  COVID-19 symptoms and resources
  Symptoms most common among patients with chronic kidney disease

**Self-care activities**

- Setting biweekly goals from a list of pre-existing goals or custom-made goals
- Tracking and monitoring the following health indicators:
  Blood pressure
  Weight
  Blood glucose
- Tracking physical and mental health symptoms including SARS-CoV-2–related symptoms, with a message of when it is recommended to contact their care provider
- Tracking flu and COVID-19 vaccinations
- Tracking and monitoring physical activity (not yet implemented)

**Adherence to medication and treatment regimens**

- Medication tracker and alarm; recommend including all medications currently taking prescribed by all providers

**Self-care self-efficacy**

- Periodic synchronous support group meetings with a focus on education regarding chronic kidney disease facilitated by a provider
- Color-coded reward system (that is linked with health indicators that they tracked) for meeting their biweekly goals (this feature is feasible for the list of pre-existing goals that could be linked to the health indicators)

## Discussion

### Principal Findings

This study described the iterative process of the development of a mobile app to facilitate self-management of nondialysis-dependent CKD. To our knowledge, our study was the first to describe the process of building a mobile app specific for patients with CKD and is guided by individuals with clinical and methodological expertise. Our stakeholder and production team included patients and providers from the entire spectrum of CKD care, along with health services and bioinformatics researchers. We used an interactive process among the research team, software team, and the stakeholders to elicit preferences and identify the requirements for the mobile app. Our stakeholders identified themes around the barriers and facilitators of CKD self-management, along the themes of CKD self-management of health care domains, which informed the features and content of our app. Our stakeholder patients and providers spoke recurrently about the importance of making the app visually appealing and user-friendly if patients were going to use it. Our findings are overall congruent with a systematic review of information technology solutions used in chronic self-management programs, which revealed that successful solutions included the following key components: education, monitoring, collaboration, and goal-setting [37].

Our findings showed that patients with CKD need a mobile app that is unique for multiple aspects of disease self-management. Unlike other chronic diseases, CKD is usually accompanied by several comorbidities such as diabetes, hypertension, obesity, and heart disease and a mobile app for CKD must address these multiple facets of disease self-management. Several themes emerged from our data that further justifies the need for the CKD app. First, patients need education during the early stages of CKD to prevent disease progression. Second, education and communication with providers need to continue outside of brief clinical encounters to help patients retain information and utilize education on disease self-management. Finally, interventions to improve the health of patients with CKD should include access to MNT. Currently, multiple mobile apps for CKD care management are available and have been previously reviewed [31]. Most existing apps for CKD management were not designed by patients with CKD and their providers, which is consistent for most mobile apps for other chronic diseases [38]. In addition, most mobile apps for CKD do not address both clinical care and health behavior promotion via motivational feedback, goal-setting, or interaction with providers [31].

Our CKD app could be used to enhance both clinical care and health behavior promotion. First, the CKD app provides a mechanism whereby biometric data, medication use, and self-reported symptoms can be tabulated in a report that can be printed or directly uploaded to the electronic health record. Alternatively, patients can simply bring their phone to a clinic visit so that providers may review data on the CKD app with the patient. During a face-to-face or video visit, providers may also work with patients to help them set goals on the app, such as keeping blood pressure at a defined target or losing or maintaining weight over a set period of time. The CKD app may also promote healthy behaviors by providing feedback on biometric data and alerting patients when symptoms may need urgent discussion with a provider. The CKD app also has a webinar function to facilitate group education or peer support.

In subsequent studies, we will examine the acceptability and usability of the app among patients with early and advanced-stage CKD, and their providers, the impact of using the app on behavioral and clinical outcomes and examine various strategies to integrate the mobile app in the clinical workflow. There is an increasing interest in shared decision-making among patients, payers, and politicians, which was codified by provisions to promote the adoption of decision aids in the 2010 Affordable Care Act [39]. Decision aids help patients become active partners in medical decision-making and include products such as educational booklets, tutorials, and mobile apps.

### Strengths and Limitations

The strengths of our study include the use of focus groups and in-depth interviews with patient and provider stakeholders to solicit multiple viewpoints. One of the limitations of our study was the use of small and convenient sample of participants, which may have limited the generalizability of our findings. Subsequent studies among patients with CKD, caregivers, and care providers recruited from a variety of settings would be necessary to assess the acceptability and usability of the mobile app.

### Conclusions

Nearly all therapies aimed at preventing kidney disease progression, and decreasing associated complications relies heavily on patient self-management, including recommendations for adherence to medication regimens [40-42], avoidance of further nephrotoxic insults [43] and maintenance of a kidney-friendly diet [44]. A mobile app that integrates domains of clinical care and health behavior promotion may be useful to facilitate CKD self-management, and our mobile app was developed in close collaboration with stakeholders in CKD. Future studies are required to examine the value of the mobile app for CKD self-management.

## Abbreviations

CKD: Chronic Kidney Disease
LUMC: Loyola University Medical Center
MNT: medical nutrition therapy
SMART: Specific, Measurable, Achievable, Realistic, and anchored within a Time Frame
